# The Last Taboo: Opening the Door on the Global Sanitation Crisis: Toilets that Make Compost

**DOI:** 10.3201/eid1509.090668

**Published:** 2009-09

**Authors:** Andi L. Shane

**Affiliations:** Emory University School of Medicine, Atlanta, Georgia, USA

**Keywords:** Sanitation, toilet, international health, book review

The publication of each of these texts during 2008—declared the International Year of Sanitation by the United Nations General Assembly to draw attention to the 2.6 billion people without access to basic sanitation—is timely. Each seeks to provide practical information and guidance to increase the proportion of the population with access to sanitation.

Maggie Black is a writer who focuses on social development and sanitation, and Ben Fawcett is an environmental health engineer. In 7 chapters, they collaborated to provide a comprehensive overview of the history of the disposal of human waste, discuss how urbanization contributed to the sanitation crisis and the lack of response from local governments, describe programs that have developed with local support to address sanitation, detail the development and design of toilets, describe social marketing efforts to increase acceptance and use of toilets and their counterparts, detail the economic implications for sanitation programs, and outline a rationale for continuing to discuss a topic formerly been regarded as taboo.

Whereas Black and Fawcett devote ≈30 pages to a discussion of toilet design and models, Peter Morgan devotes his entire 100-page book to a hands-on discussion of the design of toilets that make compost. Using pictures and graphics, he describes toilets that could be easily reproduced by the novice at home and provides economically feasible designs for toilets that produce compost. Morgan suggests that the previously regarded “nuisance” human byproduct of toilet use can be used to fertilize vegetable gardens and other vegetation. Morgan proposes designs from simple to complex and details methods to reduce insects and odors, which are common annoyances associated with composting. In the book’s introduction, he states, “It is possible to grow a tree directly in the filled toilet pit if it is planted in a layer of soil placed above the compost.” He proceeds, “So the simple toilet can have many valuable uses, in addition to being a safe way to dispose of excreta.”

Although the notion of eating a fruit or vegetable grown in excreta is unappealing to some, for most of the world’s population, a solution to both the sanitation and waste-disposal conundrums results from building a toilet that has composting ability. Black and Fawcett’s historical perspective on sanitation alludes to the potential environmental and economic implications of the benefit of such a strategy.

Each book is a worthwhile read for anyone interested in water and sanitation or in international health. Whereas Black and Fawcett’s book is likely to appeal to a wider audience, Morgan’s practical do-it-yourself approach is likely to especially interest persons involved in sanitation engineering. All 3 authors would have been delighted by the questions I received in airports, meetings, and transit while reading their respective books. The titles and topic remain an area of interest and fascination.

